# Supplementary material for: Are carbon emissions trading and green financial instruments synergistic? -Comprehensive quantitative research based on content analysis
Source: PLoS One. 2024 Mar 7;19(3):e0298601. doi: 10.1371/journal.pone.0298601 (PMC10919601; doi:10.1371/journal.pone.0298601)
Supplement: S1 Data — (ZIP) [file pone.0298601.s001.zip › Data/地级市Z_F工作报告.zip!/╔╜╢1⁄2/═■║ú╩╨/└┤╫╘╣1⁄2╓┌║┼í╛┬φ┐╦╩2╛▌═°í┐_2014.docx]

各位代表：

　　现在，我代表威海市人民政府向大会报告工作，请予审议，并请各位政协委员和其他列席人员提出意见。

一、2013年政府工作回顾

　　过去的一年，在省委、省政府和市委的坚强领导下，市政府和全市人民一道，全面贯彻党的十八大和十八届二中、三中全会精神，认真落实省第十次党代会、市第十四次党代会精神，牢牢把握稳中求进的工作总基调，紧紧围绕现代化幸福威海建设，坚持生态立市、科教兴市、产业强市，着力稳增长、调结构、促改革，扎实做好各方面工作，经济社会发展稳中有进。全市生产总值增长10.8%，公共财政预算收入增长13.4%，城镇居民人均可支配收入增长9.8%，农民人均纯收入增长11.6%，居民消费价格涨幅控制在1.5%，城镇登记失业率控制在1.52%。

　　（一）坚持产业强市，大力推进转方式调结构。强化工业带动，突出技术改造和技术创新，着力推进传统产业转型和战略性新兴产业成长。实施省级战略性新兴产业项目27项，新增规模以上工业企业99家，新认定科技型中小企业27家，工业技改投资增长20%，工业增加值增长11.7%。促进农业提质增效、农民持续增收，新增农超对接基地3.5万亩、农民合作社551家、现代果园6万亩，推广测土配方施肥157.5万亩，粮食产量保持稳定。大力发展海洋产业，新增远洋渔船128艘，远洋捕捞产量增长50%，海洋产业增加值增长10.5%。积极培育服务业新型业态，新增大型商业设施42万平方米，社会消费品零售总额预计增长13%左右；推进旅游标准化建设，旅游总收入增长13.3%；港口货物吞吐量达到7001万吨，增长13%；开通至台北的直线航班，机场旅客吞吐量首破百万，达到114.6万人次，增长25.6%；金融机构存贷款余额分别增长15.4%和13.4%。服务业增加值预计增长11%左右，三次产业比重由7.7：53.4：38.9调整为7.6：51.8：40.6。组织实施百项市级重点项目，37个项目建成投入使用，固定资产投资预计增长20.5%左右。

　　（二）坚持科教兴市，增强区域创新能力。按照“出政策、搭平台、增投入”的思路，加大对企业科技创新的扶持力度，市级财政科技支出增长30.6%。新建科技企业孵化器10家，创建省级以上企业研发平台16家，实施产学研合作项目82项，发明专利授权量预计增长18%。启动智慧城市建设，建成云计算中心和工业设计、公共服务云平台。强化教育基础保障，新建公办及公办性质幼儿园10所，新增农村中小学新模式校车273辆，新建远程录播教室40个、交互式多媒体教室1084个，建成4个职业教育示范实训基地，三市一区全部通过国家义务教育发展基本均衡县验收，成为全省唯一实现“满堂红”的市。实施重点人才工程，新建大学生创业孵化基地5家，引进各类人才2.7万人，增长53%。

　　（三）坚持城乡统筹，推进市域一体化发展。出台城市化发展纲要，在户籍管理、公共服务、住房保障等方面推出一系列政策措施，推动农业转移人口市民化。重点区域规划编制完成，实施基础设施项目59个、产业项目54个，完成投资182亿元。工业新区升级为国家级临港经济技术开发区。蓝色经济区建设14个重大事项进展顺利，4个园区成为全省首批海洋特色产业园。实施36项城建重点工程，青荣城际铁路工程快速推进，江家寨立交桥、威海港国际客运中心投入使用，统一路南延工程竣工通车。扎实推进社区建设，城市社区全部建立新型组织体系。

　　（四）坚持改革开放，不断激发市场活力。按照简政放权的要求，推动行政服务提速，着力优化营商环境。启动建设工程项目审批模块化流程再造，推动串联审批向并联审批转变。清理行政事业性收费项目138项，减轻企业和社会负担6000万元。强化金融服务创新，新引进各类金融机构10家，社会融资规模达到284.9亿元，是上年的2倍。有序推进农村三项改革，完成土地确权登记685个村、集体产权制度改革135个村，新增土地流转面积15.5万亩。积极拓展对外开放新格局，成为全国首批中欧城镇化伙伴关系合作示范城市，与荷兰阿克马市、比利时根特市缔结友好城市关系。实际到账外资9.2亿美元，实际利用内资554亿元，均增长15%。外贸进出口总额达到171.5亿美元，其中出口107亿美元，分别增长0.2%和0.5%。

　　（五）坚持生态立市，切实加强环境保护。组织开展生态安全格局、城乡建设用地、公共开放空间布局等规划编制工作。健全水资源利用、海岸带管理、沙滩保护等方面的管理制度。完善大气污染联防联控工作机制，开展散流物体运输、弃土堆、燃煤小锅炉、机动车尾气等专项整治，空气质量指数保持全省首位。开展环境安全隐患排查。实施刘公岛、九龙湾等12个海洋整治修复项目，完成7条河道综合治理。成片造林10万亩。万元生产总值能耗预计降低3.7%，主要污染物排放量继续下降。成功创建首批国家级海洋生态文明建设示范区。率先通过省级生态市验收，三市全部成为国家生态市。

　　（六）坚持民生为重，扎扎实实为群众办实事。健全民生保障机制，民生支出占财政总支出的比重达到60.7%，27项民生实事基本完成。强化创业就业服务，新增城镇就业4.4万人，转移农村劳动力2.4万人。完善社会保障制度，城乡居民基础养老金标准、医疗保险待遇和最低生活保障标准都有新提高。实施十项助残工程，帮助2.5万名残疾人解决实际困难。完善社会化养老服务体系，新建养老机构19处，新增机构养老床位2419张。加强公共租赁住房建设，新增各类保障性住房3790套。完成16个旧生活区和270个村的环境综合整治。实施供热计量和建筑节能改造1008.9万平方米。购置节能环保型公交车230辆，启动智慧交通建设，智能化改造公交线路26条，改善了市民出行条件。提高医疗卫生服务能力，创建3家三级甲等医院，新建农村标准化卫生室179处、城市社区卫生服务中心3处。实施文化惠民工程，完善提升400个农村文化大院服务功能，免费送戏下乡503场。新建农民体育健身工程495处，实现村级全覆盖。实施餐饮单位厨房亮化和远程电子监控工程437处，创新监管模式保障群众饮食安全。

　　（七）坚持统筹兼顾，协调发展各项社会事业。积极创建全国文明城市。圆满完成十艺节“群星奖”戏剧类比赛承办任务，被评为文化强省建设先进市。成功举办第七届国际人居节和国际长距离铁人三项赛等活动。竞技体育实现新突破，在第十二届全运会中取得12金、12银的历史最好成绩。加强平安威海建设，被评为首批全国法治城市创建先进市。健全安全生产监管制度，建立专职消防员队伍，完成全市205处应急避难场所建设。国防动员、人民防空、民兵预备役工作进一步加强，人口计生、民族宗教、妇女儿童、外事、侨务、对台、档案、史志、地震、气象、仲裁、慈善等事业取得新进步。

　　（八）坚持依法行政，加强政府自身建设。扎实推进法治政府、服务型政府、廉洁政府建设，深化机关作风建设年活动，切实规范行政行为。加强政府信息公开，完成65个部门的行政权力公开，各级政府和部门开通政务微博81个，建立网络监督员制度，及时回应群众关切。自觉接受人大的法律监督、政协的民主监督，主动听取各民主党派、工商联、各人民团体和人民群众的意见建议。办理人大代表建议和政协委员提案228件，办复率达到100%。强化监察审计等内部监督，开展廉政巡回讲堂活动，扎实推进廉政建设。加强行风评议工作，深入开展“庸懒散”专项治理，机关作风有新的转变。

　　各位代表！政府工作的每一份成绩，都离不开全市上下的共同努力，离不开社会各界的大力支持。在此，我代表市政府，向全市人民，向人大代表和政协委员，向各民主党派、工商联、各人民团体和各界人士，向驻威人民解放军、武警官兵，向各位老领导、老同志，向所有关心支持威海发展的海内外朋友，致以崇高的敬意和衷心的感谢！

　　在总结成绩的同时，我们也清醒地认识到，工作中还存在一些困难和问题。主要表现在：产业结构不够优化，传统产业转型升级压力增大，战略性新兴产业尚未形成规模，服务业比重仍然偏低，科技创新能力不强，转方式调结构任务艰巨；部分企业经营困难，产业投资特别是制造业投资不足，消费需求增速放缓，经济企稳回升的基础还不牢固，经济运行存在下行压力；外部需求依然低迷，进出口增长乏力，贸易结构有待优化，园区的综合配套能力还不够强，对经济增长的贡献度有待提高，开放型经济发展亟需谋求新突破；部分减排工程进展缓慢，节能减排压力加大，生态环境优势需要进一步巩固和提升；民生领域的一些问题尚未妥善解决，影响社会和谐稳定的一些矛盾依然突出；个别机关工作人员懒散懈怠，少数行政机关和公共服务单位的办事效率有待提高，廉政建设有待加强。对此，我们一定认真对待，努力加以解决，切实改进政府工作。

二、2014年政府工作的目标任务

　　各位代表！当前我市正处于改革创新的攻坚时期，威海发展面临突破提升、转型跨越的历史任务。我们要顺应人民群众对美好生活的新向往，抢抓全面深化改革的历史机遇，以改革创新的精神、思路、办法来谋划发展全局，突破制约威海长远发展的瓶颈问题，在新一轮竞争的大格局中找准位置，赢得工作主动权，再创科学发展新优势。

　　2014年政府工作的总体要求是：全面贯彻落实党的十八大和十八届二中、三中全会精神，以邓小平理论、“三个代表”重要思想、科学发展观为指导，牢牢把握稳中求进的工作总基调，把改革创新贯穿于经济社会发展各个领域各个环节，坚持生态立市、科教兴市、产业强市，推动工作指导重大转变，打好转方式调结构攻坚战，着力激发市场活力，努力提高经济发展质量和效益，切实保障和改善民生，提高社会治理能力，促进经济持续健康发展、社会和谐稳定，推动全域城市化、市域一体化发展，加快建设现代化幸福威海。

　　主要预期目标为：生产总值增长10%，公共财政预算收入增长13%，固定资产投资增长20%，社会消费品零售总额增长13%，外贸进出口增长5%，实际利用外资稳定增长，实际利用内资增长15%，城镇居民人均可支配收入、农民人均纯收入均增长11%，居民消费价格涨幅控制在3.5%左右，城镇登记失业率控制在2%以内，全面完成省下达的年度节能减排约束性目标。

　　工作中，要以党的十八届三中全会和习近平总书记系列重要讲话精神为指引，坚持以解放思想为先导，坚定不移地推进科学发展，加快工作指导转变。一是把推动发展的立足点切实转变到以提高经济发展质量和效益为中心上来。牢固树立质量效益优先的工作导向，更加注重全面协调可持续发展，让经济发展的质量更高、效益更好、后劲更足、步伐更加坚实。二是把推动发展的着力点切实转变到转方式调结构上来。坚持凤凰涅槃、腾笼换鸟的思路，千方百计做强存量、做大增量、做优总量，主动谋求转型发展，让威海的开放优势更加突出，产业结构稳步优化，发展方式加快转变。三是把改革创新贯穿于经济社会发展各个领域各个环节。紧紧围绕使市场在资源配置中起决定性作用和更好发挥政府作用，正确推进改革、准确推进改革、有序推进改革、协调推进改革，让体制机制更加顺畅，营商环境更加优越，创新创业活力竞相迸发。四是把保护环境的理念落实到经济社会发展的各方面和全过程。牢固树立“绿水青山就是金山银山”的理念，实施最严格的环境监管，推动最集约的资源利用，开展最积极的生态建设，让威海的天更蓝、水更清、山更绿、空气更清新。五是把保障和改善民生作为推动发展的根本目的。牢固树立群众观念，用更加开放的态度加强政民沟通、了解社情民意，以群众看得见、感受得到的工作实绩回应群众的期盼和要求，让全市人民得到更多的改革红利，享受更多的发展成果。六是把优化行政服务作为推动改革发展的重要保障。顺应改革发展的新要求，进一步简政放权，做好“接、放、管”的文章，推动政府管理方式由事前审批向事中事后监管转变，由被动管理向主动服务转变，切实规范政务运行，推动行政服务提速，有效调动各方面的积极性，汇聚起干事创业的强大正能量。

三、2014年政府工作安排

　　（一）推动经济转型升级。坚持产业强市，按照一产抓特色促跨越、二三产抓结构上规模的思路，着力拉长产业链条，培植优势产业集群，推动三次产业在更高层次上协调发展，产业内部结构从价值链低端向高端加快转变。

　　强化工业带动。实施产业集群“3+4”发展战略，培育膨胀新信息、新医药与医疗器械、新材料及制品3个战略性新兴产业集群，改造提升机械制造、食品加工、运输设备、纺织服装4个传统优势产业集群。实施龙头企业扩张、高成长企业提升、新兴企业培育“三百工程”，推动各个行业的骨干企业加速膨胀，形成示范带动效应。推动“个转企、小升规、普升高”，新增规模以上工业企业100家、高新技术企业18家。引导企业加大技术改造力度，淘汰落后产能，化解过剩产能，提高技术装备水平，增强市场竞争力。引导骨干企业突出技术含量高的核心生产环节，将辅助生产环节向中小微企业转移，促进产业分工协作，提高产业集群度。力争工业增加值增长11.5%。

　　壮大现代服务业。在巩固提升传统服务业的基础上，突出生产性服务业、信息消费产业、新型生活服务业三大板块，大力培育服务业新型业态, 推动服务业结构调整和增量扩容。推动制造业向价值链前端的研发、设计、规划、品牌和后端的销售、服务、融资、营销、广告等环节延伸，催生更多的商务服务企业和衍生产业，促进服务业与制造业良性互动。加大各类金融机构的引进力度，丰富金融市场层次和产品，稳步扩大金融业整体规模。加强临港物流园区建设，培植壮大龙头物流企业，鼓励发展第三方物流，扶持冷链物流业发展。推进国家信息消费试点城市建设，支持企业创新商业模式，发展新兴信息服务、电子商务、数字文化等信息消费产业，信息消费增长10%以上。完善消费鼓励政策，构建扩大消费需求长效机制，着力发展养生养老、健康服务、家庭服务、旅游、文化、体育等新型生活服务业。制定文化产业发展规划，繁荣活跃文化市场，引导扩大文化消费。深入推进旅游标准化建设，创新营销方式，加强旅游市场开发，加快旅游公共服务中心、集散中心建设，旅游总收入增长12%以上。力争服务业增加值增长11.5%，占生产总值比重提高1个百分点以上。

　　促进农业提质增效。大力发展高产、优质、高效、生态、安全农业，加快农业发展方式转变，促进农业增效、农民增收。坚持和完善农村基本经营制度，大力扶持家庭农场、专业大户、农民合作社、龙头企业等新型农业经营主体，深化供销社改革，构建现代农业经营体系。完善农业技术服务体系，推广机械化保护性耕作技术，扩大智能测土配方施肥服务范围，加强酸化土壤治理，推进小型农田水利重点县项目建设，增强农业综合生产能力，稳定粮食产量。加强农业投入品管理，健全农产品质量和食品安全追溯体系，推进中韩国际食品安全示范区试点工作，争创国家农产品质量安全监管示范市。继续推进农超对接，拓宽农产品销售渠道。推动一产向二产三产延伸，提升果业、西洋参、水貂、苗木花卉等特色农业发展层次，带动关联产业发展。

　　大力发展海洋经济。深入推进蓝色经济区建设，着力培育现代海洋产业体系，推动渔业大市向海洋经济强市跨越。加快发展海洋生物产业，积极开发海洋生物保健品、新型海洋药物、海洋冷冻调理食品等新产品，提高海产品增值水平。推进海洋生物遗传育种中心建设，做强做优种苗产业。推进海外综合性远洋渔业基地建设。加快发展休闲渔业，建设40处休闲渔业示范基地。大力培育临港机械装备、海洋仪器装备等海洋装备制造业，打造专业性现代海洋装备及配套制造业基地。推动造船业优化重组、转型升级、特色发展。加快推进16个蓝色经济区建设重大事项，争创国家海洋高技术产业基地。

　　强化重点项目建设。发挥投资的关键作用，促进投资稳定增长和结构优化。今年组织实施133项市级重点项目，年内计划投资452.1亿元；实施100项重点工业项目，年内计划投资240亿元；实施36项城建重点工程，年内计划投资263.9亿元。推动青荣城际铁路建成通车，加快荣文高速公路、文莱高速公路、石烟线外移、威海机场跑道改造、威海湾港区航道扩建等项目建设。推进金线顶、九龙湾等区域整体开发改造，建成市区综合交通枢纽、刘公岛客运中心。开工建设泊于水库，推进南水北调续建配套工程建设。实施苘山净水厂二期、经区污水处理厂搬迁改造等供排水工程。积极创新投融资方式和渠道，更多利用市场化手段推进基础设施建设，解决制约我市长远发展的瓶颈问题。

　　加快智慧城市建设。坚持以信息化提升工业化，推广应用工业设计云平台，为企业提供软件、技术和信息外包服务，培育以物联网、云计算为基础的智慧产业。探索建立跨境电子商务推进机制，支持专业电子商务平台建设，打造有影响力的威海企业网上集聚区。推进电子政务云平台建设，拓展公共服务云平台应用领域，实施智慧交通、智慧旅游等示范项目，让公众享受智慧服务带来的便利。

　　（二）支持全社会创新创业。坚持科教兴市，激励和保护各类群体的创新创业积极性，让创新创业成为发展的强力引擎。

　　加强创新平台建设。强化创新驱动，突出企业主体地位和市场导向作用，引导和扶持企业创新平台、公共创新平台、科技企业孵化器建设，新建省级以上企业研发平台9家，集聚科技创新资源。发展技术交易市场，建设科技创新综合服务平台，推动大型科学仪器开放共享。健全扶持政策和考核办法，强化科技企业孵化器功能建设，探索建立专业孵化器，新增科技孵化面积4万平方米。支持建立以企业为中心的产学研协同创新机制，培育产学研合作示范企业、示范基地、创新团队和创新联盟，提高产学研合作实效。

　　健全集聚人才机制。强化人才支撑，完善海内外高层次人才引进政策，推进高端人才交流与合作项目，新建10个引智成果示范推广基地，实施68个引智项目。实施“导师带徒”工程、专业技术人员知识更新工程和技能振兴计划，为各类人才成长提供支持。完善人才评价激励机制，促进人才顺畅流动，让各类人才都有施展才华的广阔天地。

　　推动科技金融结合。发挥政策导向作用，鼓励企业加大科技创新投入，力争全社会研发投入占生产总值的比重提高0.1个百分点。探索建立科技扶持资金“拨改投”使用方式，提高资金使用效率。设立创新投资引导基金，吸引各类社会资本投资种子期、初创期科技型企业。扩大科技支行试点范围，助推科技型小微企业快速成长。

　　优化创业服务。推进工商注册制度便利化，减少工商登记的前置许可事项，放宽企业名称使用、经营场所、注册资本等条件，扩大个体工商户免登记范围。加强中小微企业服务体系建设。开展“快易贷”行动，切实发挥创业小额担保贷款政策作用。推动省级创业孵化基地和大学生创业孵化基地建设，用好创业带动就业专项资金，为各类创业群体提供差异化专业服务。

　　实施积极的就业政策。加强就业培训和就业援助，促进以高校毕业生为重点的青年就业和农村转移劳动力、城镇困难人员、退役军人就业，新增城镇就业3.6万人，转移农村劳动力1.4万人。创新劳动关系协调机制，规范劳务派遣管理，构建和谐劳动关系。健全最低工资和工资支付保障制度，完善企业工资集体协商制度，促进居民收入持续增加。

　　（三）全面深化改革。贯彻落实中央和省全面深化改革的统一部署，按照胆子要大、步子要稳的要求，积极有序推进改革。

　　理顺市场监管机制。抓住市场化这一核心，推动建设统一开放、竞争有序的市场体系。调整工商、质监、食品药品监管体制，改革企业年检制度，推动企业巡查制度向抽查制度转变。健全社会征信系统，构建中小企业信用评价和信用信息共享服务机制。

　　完善国有资产监管制度。以管资本为主加强国有资产监管，推进股权多元化改革，积极发展混合所有制经济。完善国有资本经营预算制度。调整理顺国有企业负责人管理体制，积极引入市场化选聘机制，健全经营业绩考核与薪酬管理制度。探索建立公益设施市场化运作模式，管好用活文化、体育等公益设施。

　　深化财税管理体制改革。加快构建全面规范、公开透明的预算制度，扩大财政预决算和“三公”经费公开范围。加强税收征管，严格控制非税收入增长，提高财税收入质量。深入开展“营改增”试点。推进会计集中核算向国库集中支付制度转轨。把地方政府性债务分类纳入全口径预算管理，严格政府举债程序，严控政府举债规模，严禁违规担保、过度举债、隐性举债。落实偿债准备金制度，健全债务风险预警机制，防控地方债务风险。

　　推动金融改革创新。推进民营银行试点工作，鼓励小额贷款公司发展，规范设立民间资本管理机构、民间融资登记服务机构。做好知识产权质押融资试点工作。加强上市后备资源培育，推动更多符合条件的企业加快上市进程。积极利用“新三板”，拓宽科技型企业直接融资渠道。建立小微企业贷款风险补偿机制，设立资金池，缓解小微企业融资难。健全地方金融监管体制，优化金融生态环境，防范区域性、系统性金融风险。

　　健全城乡发展一体化体制机制。积极争取全国中小城市综合改革试点。在示范镇开展农村综合改革试点工作。积极稳妥推进农村三项改革。加强基层民主政治建设，做好村居两委换届选举工作。有序推进农业转移人口市民化，稳步实现城镇基本公共服务常住人口全覆盖。

　　加快生态文明制度建设。实行最严格的源头保护制度、损害赔偿制度、责任追究制度，完善环境治理和生态修复制度。强化节能节地节水、环境、技术、安全等市场准入标准，设定行业禁入线，拒绝高耗能、高污染、高风险和资源性项目落地。深化资源性产品价格改革，健全价格联动机制，完善居民生活阶梯价格制度。

　　推进社会事业改革创新。推行中小学校长职级制改革，建立学校间校长教师交流轮岗制度。出台初中学业水平考试改革方案。扩大职业教育学历证书与职业资格证书“双证互通”试点。统筹推进医疗保障、医疗服务、公共卫生、药品供应、监管体制综合改革，做好公立医院综合改革试点工作，完善网络化城乡基层医疗卫生服务运行机制，健全全民医保体系。探索推动图书馆、博物馆、群众艺术馆等公共服务机构组建理事会。

　　加快转变政府职能。以建设工程项目审批流程再造为突破口，正式推行模块化审批模式。引入竞争机制，推动行业协会、中介组织与行政机关真正脱钩。推行公开承诺制，规范公共服务单位和中介组织的服务行为，清除行政审批提速障碍。完成市县政府机构改革。积极稳妥推进事业单位分类改革。建立政府向社会力量购买服务制度，把适合采取市场化方式提供的公共服务，逐步交由社会力量承担。

　　（四）增创开放型经济新优势。借力中欧、中韩合作的新机遇，推动对内对外开放相互促进、引进来和走出去更好结合，谋求开放工作的新突破。

　　推进重点园区建设。建立中欧合作长效工作机制，全面加强与欧洲在产业、环境、科技等领域的战略合作。推进中韩（威海）经济合作示范区建设，探索中韩陆海联运新机制，打造韩国食品日用品交易集散中心，搭建中韩产业合作平台、商品交易平台。加快临港经济技术开发区集约集聚发展，积极推动威海出口加工区转型升级为综合保税区，推动威海南海新区上档升级，加强各类园区配套建设和服务，提高开发层次和发展水平。

　　增强招商引资实效。围绕培植产业集群、拉长产业链条，有针对性地开展产业招商、定向招商，引进骨干龙头企业、核心配套项目。利用好各类招商平台，瞄准目标企业、重点区域，实行小分队招商，提高招商成功率。落实招商引资主体责任，完善外资项目事前事后评价办法，注重考核招商实效和真实投资强度。

　　加强对外经济贸易合作。按照巩固传统市场、拓展新兴市场、开拓国内市场的思路，组织实施“百展市场开拓计划”，办好国际食品博览会、国际渔具博览会等展会活动，促进内外贸一体化发展。指导优势企业进入联合国采购体系、优势产品进入国家援外物资采购目录。大力发展服务外包和服务贸易。推动加工贸易企业转型发展。支持企业“走出去”，设立境外研发中心、技术中心、设计中心，布局跨国产业链，提升跨国经营能力。

　　促进对外贸易便利化。深化通关业务改革，加快电子口岸建设，推进通关作业无纸化、区域通关等便利措施，推动关检合作，实施查验单位一个平台查验、放行模式，进一步优化通关作业流程，减少环节，降低成本，提高通关效率。

　　（五）促进市域一体化发展。紧紧围绕提高新型城镇化发展质量，强化重点区域带动和示范镇建设，加快推进以人为核心的城镇化。

　　加快重点区域开发建设。坚持“政府主导、社会参与、市场运作、城市融合”，突出产业招商和项目建设，引导各类资源向重点区域聚集，产业项目向重点区域布局，有序推进重点区域开发建设。合理布局生态生产生活空间，科学规划路网、管网，提高重点区域规划建设水平。统筹推进示范镇建设，推动县域联动发展，提升县域经济发展水平。

　　加强城市建设管理。强化核心区域和重点片区规划控制，做好各类规划的统筹衔接。完善城市社区网格化管理模式，扩大城市综合管理信息系统应用范围，提升城市精细化管理水平。购置290辆节能环保型公交车，扩大公交覆盖范围，改善市民乘车环境。加强城市地下管网隐患排查与治理，确保安全运行。加强老洋房、海草房等文化遗产保护与利用，延续城市历史文脉。

　　推进农村新型社区建设。强化以城带乡，促进基本公共服务均等化。编制农村新型社区布局发展规划，年内建设新型社区15个，补助建设改造农房1.2万户。完成所有村居便民服务代办点建设。加强镇村基础设施建设，实施农村规模化供水工程，铺设供热管道31.5公里、燃气管道108.6公里，改造农村公路194公里，建成电气化村37个，建设农村标准化卫生室218处，让农村居民享受更好的公共服务。

　　（六）加强生态文明建设。坚持生态立市，牢固树立保护生态环境就是保护生产力、改善生态环境就是发展生产力的理念，强化环保、安全标准硬约束，集中开展专项治理行动，依法严惩超标排放，确保完成节能减排目标任务，促进生态生产生活“三生”共融。

　　开展大气污染防治行动。强化市域一体监管，健全空气质量监测网络和实时发布制度，实施重点领域节能工程，加强重点燃煤企业脱硫脱硝设施改造和运行管理，全部拆除燃煤机组脱硫旁路，整治集中供热范围内的燃煤小锅炉，治理异味超标排放企业，加强散流物体运输、弃土堆等扬尘污染治理，加快淘汰黄标车，控制机动车尾气排放，进一步提高空气质量。

　　开展水生态专项治理行动。严格执行用水总量控制、用水效率控制、水功能区纳污控制三条红线，严厉查处排污倒废等环境违法行为，加强农业面源污染治理，提高镇村污水集中处理能力，推进水系生态保护与修复工程，加强湿地、地下水、地热水和饮用水水源地保护，积极争创省级水生态文明城市。

　　开展海洋环境专项整治行动。加强海岸带执法巡查，强化海域用途管制，规范海域使用权转让出租行为，坚决制止违法违规填海，严厉查处盗挖海砂、非法养殖、乱搭乱建、违规排污等行为，实施威海湾等13个海域海岛海岸带生态修复项目，推进小石岛、海西头等海洋公园和海洋保护区建设，保护好宝贵的岸线、沙滩、海域和海岛。

　　开展土地资源清理整治行动。严格落实耕地保护目标责任，实施市级投资土地开发整理项目14个，新增高标准基本农田24万亩。扎实推进土地增减挂钩和工矿废弃地复垦调整利用，加强闲置和低效利用土地处置工作。实行差别化供地政策，节约集约利用土地。

　　开展城乡环境综合整治行动。坚持整治与管护并重，推进255个村的环境综合整治，对已完成整治的村安排管护经费补助。中心城区实施19个旧生活区环境综合整治工程。开展餐厨废弃物综合整治。完成275家规模化养殖场污染治理。加强森林防火和病虫害防治工作，大力实施绿化工程，成片造林7.5万亩。

　　（七）保障和改善民生。坚持把人民群众对美好生活的向往作为奋斗目标，切实解决好群众最关心、最直接、最现实的利益问题，努力为社会提供多样化公共服务，更好满足人民群众需求。

　　坚持教育优先发展。推动义务教育优质均衡发展，继续实施教育信息化三年行动计划，积极利用信息化手段扩大优质教育资源覆盖面。深入实施素质教育，启用市中小学生综合实践基地，培养学生实践能力和创新精神，标本兼治减轻学生课业负担；加强体育卫生艺术教育，为36所学校建设塑胶跑道和运动场地，促进学生身心健康、体魄强健。推动学前教育公益普惠发展，公办及公办性质幼儿园比例达到67%。深化产教融合、校企合作，推进市属职业学校资源整合，规范职业教育发展。加强与驻威高校共建，推进北京交通大学威海校区、山东交通学院威海校区、威海海洋职业学院的建设与招生工作。强化校园、校舍、校车安全管理。加强师德师风建设，提高教师队伍素质。

　　健全社会保障体系。启动实施城乡一体的居民基本医疗保险制度，完善居民基本养老保险制度，稳妥做好被征地农民养老保障工作，提高社会保障绩效。发展老年服务产业，开工建设市社会福利中心，新建129个农村幸福院，新增机构养老床位1200张。扩大高龄津贴发放范围，提高补贴标准。加强困难群众救助工作，提高城乡居民最低生活保障标准。完善残疾人医疗保险、养老保险、居家护理等补贴政策，推进残疾人奔小康工程。改进住房公积金归集、使用、监管机制。推动公共租赁住房和廉租住房并轨运行，新建公共租赁住房1200套。

　　提高医疗卫生服务水平。完善重大疾病防控和突发公共卫生事件应急处置机制，增强急重症患者转运及急救能力。开工建设市立医院全科医生培训基地，扩建改造市妇女儿童医院。实施基层中医药服务能力提升工程。组织开展医疗市场集中整顿，加强公立医院监管。强化医德医风建设，构建和谐医患关系。健全医患纠纷第三方调解机制。适时调整生育政策，完善计划生育利益导向机制，开展计生助福行动，关怀计划生育特殊家庭。

　　加强公共文化服务。健全公共文化服务体系，开工建设市图书馆新馆和群众艺术馆新馆，提升“四馆一站”免费开放水平，创建省级公共文化服务示范区。办好文化惠民实事，开展“幸福威海”系列群众性文化活动，扶持国际会议中心剧院面向群众举办高水平演出，免费送戏下乡500场。加强公民道德建设，强化未成年人思想道德教育，广泛开展志愿服务活动。深入开展全国文明城市创建活动，扎实推进乡村文明行动。

　　加强公共安全保障。创新社会治理方式，完善海陆一体的平安建设工作机制。深化安全生产网格化监管，强化企业安全生产主体责任，完善安全生产专家队伍，探索建立政府购买第三方安全生产服务机制，健全隐患排查治理和安全预防控制体系，遏制重特大安全事故发生。深入开展“平安行·你我他”行动，加大事故易发路段和行车不良习惯综合整治。建立食品药品生产经营单位安全信用档案，健全最严格的覆盖全过程的监管制度，严厉打击制假售假行为，保障食品药品安全。加强高致病性禽流感等疫病防控，推进无规定动物疫病区建设。强化应急管理体系建设，加强应急教育培训和预案演练，增强突发事件应急处置和救援能力。加强社会治安综合治理，依法严密防范和惩治违法犯罪活动。健全矛盾纠纷调处化解综合机制，营造和谐稳定的社会环境。

　　协调发展各项社会事业。深化法治威海建设，完善法律援助、司法救助制度。健全社会组织孵化机制，培育公益慈善、社会福利、社区服务等类型的社会组织。完善公益性体育设施，推进市乒羽健身中心、城市健身慢行道建设，承办好长距离铁人三项世界锦标赛，广泛开展全民健身活动。组织好第三次全国经济普查。抓好双拥共建和优抚安置工作，加强国防动员、民兵预备役、人民防空和打击走私等工作。做好民族宗教、妇女儿童、外事、侨务、对台、仲裁、档案、史志、气象、防震减灾和对口支援工作。

　　各位代表！让政府工作更加贴近群众需求，是我们的不懈追求。根据群众意愿，结合工作实际，今年集中力量办好9大类25项民生实事。我们将积极履行承诺，切实把实事办好，努力通过政府的工作，让人民群众得到更多的实惠，过上更好的生活。

　　（八）加强政府自身建设。紧紧围绕建设法治政府、服务型政府和廉洁政府，以为民、务实、清廉的作风推动各项工作，切实增强政府公信力和执行力。

　　切实改进工作作风。扎实开展党的群众路线教育实践活动，健全改进作风常态化机制。完善直接联系和服务群众制度，健全领导干部带头改进作风、深入基层调查研究机制，帮助企业和群众解决实际问题。坚持政府带头过紧日子，严格执行厉行节约各项规定，从严从简开展公务活动，大力压缩一般性支出，严格控制“三公”经费，将财政资金更多投向民生保障和公共服务领域。

　　大力推进依法行政。加强普法教育，引导督促公务员切实增强法治意识，自觉遵纪守法，严格按照法定权限和程序行使权力。健全政府法律顾问制度，完善规范性文件、重大决策合法性审查机制。建立行政复议依法先行调解制度，规范行政复议委员会的运行机制。加强行政违法预防工作，做到严格规范公正文明执法。

　　深化行政审批制度改革。推进部门相对集中行政许可权工作，实行“两集中、四到位”，一口对外服务，现场集中办公。完善电子审批、电子监察系统，实现三级联动、联合办理和信息共享。进一步精简审批事项，简化办事流程，清理规范行政事业性收费、经营服务性收费，提高服务效率和办事透明度。

　　强化政府信息公开。健全信息公开网络，完善政府信息发布制度，充分利用行风热线、社情民意调查中心和各类新媒体，发挥群众监督员和网络监督员的作用，及时倾听群众呼声，回应社会关切。加强政府网站和政务微博运行管理，增强网上办事和互动交流功能。建设运营威海市民网，搭建为民办事、与民沟通的新平台。整合紧急救助热线、政务服务热线和便民服务热线，打造更加公开便利的便民服务和政民互动平台。

　　自觉接受各方面监督。开展财政资金管理使用绩效审计，加大对政府投资项目的审计力度，形成“花钱必问效、无效必问责”机制。加强行政效能监察，改进工作考核机制。自觉接受人大的法律监督、政协的民主监督、媒体的舆论监督、群众的社会监督，认真办理人大代表建议和政协委员提案。

　　加强政府廉政建设。全面落实党风廉政建设责任制，健全岗位风险防控制度，从源头上防治腐败。严格执行各项纪律规定，严肃查处各类违纪违法案件，坚决纠正损害群众利益的不正之风，以良好的作风和形象，赢得人民群众的信任和支持。

　　各位代表！实现让人民群众过上幸福生活的中国梦，是我们肩负的使命和责任。让我们更加紧密地团结在以习近平同志为总书记的党中央周围，在市委的坚强领导下，凝心聚力，真抓实干，谱写全面深化改革、推动科学发展的新篇章，为建设现代化幸福威海而努力奋斗！
